# Mass spectrometric imaging of brain tissue by time‐of‐flight secondary ion mass spectrometry – How do polyatomic primary beams C_60_^+^, Ar_2000_^+^, water‐doped Ar_2000_^+^ and (H_2_O)_6000_^+^ compare?

**DOI:** 10.1002/rcm.7285

**Published:** 2015-09-03

**Authors:** Irma Berrueta Razo, Sadia Sheraz, Alex Henderson, Nicholas P. Lockyer, John C. Vickerman

**Affiliations:** ^1^ Manchester Institute of BiotechnologyThe University of ManchesterManchesterM13 9PLUK; ^2^ School of Chemical Engineering and Analytical ScienceThe University of ManchesterManchesterUK; ^3^ School of ChemistryThe University of ManchesterManchesterUK

## Abstract

**Rationale:**

To discover the degree to which water‐containing cluster beams increase secondary ion yield and reduce the matrix effect in time‐of‐flight secondary ion mass spectrometry (TOF‐SIMS) imaging of biological tissue.

**Methods:**

The positive SIMS ion yields from model compounds, mouse brain lipid extract and mouse brain tissue together with mouse brain images were compared using 20 keV C_60_^+^, Ar_2000_^+^, water‐doped Ar_2000_^+^ and pure (H_2_O)_6000_^+^ primary beams.

**Results:**

Water‐containing cluster beams where the beam energy per nucleon *(E/nucleon)* ≈ 0.2 eV are optimum for enhancing ion yields dependent on protonation. Ion yield enhancements over those observed using Ar_2000_^+^ lie in the range 10 to >100 using the (H_2_O)_6000_^+^ beam, while with water‐doped (H_2_O)Ar_2000_^+^ they lie in the 4 to 10 range. The two water‐containing beams appear to be optimum for tissue imaging and show strong evidence of increasing yields from molecules that experience matrix suppression under other primary beams.

**Conclusions:**

The application of water‐containing primary beams is suggested for biological SIMS imaging applications, particularly if the beam energy can be raised to 40 keV or higher to further increase ion yield and enhance spatial resolution to ≤1 µm. © 2015 The Authors. *Rapid Communications in Mass Spectrometry* Published by John Wiley & Sons Ltd.

Mass spectrometric imaging of biological tissue and cells is being widely explored by the main desorption techniques.^1^, ^2^, ^3^, ^4^, ^5^, ^6^ Many practitioners are beginning to regard the technique as a routine methodology for determining the spatial distribution of chemistry in tissue samples. However, the related issues of molecular sensitivity and the matrix effect are severe constraints to the confident application of imaging mass spectrometry to the analysis of complex samples, especially those related to medical conditions.^7^, ^8^

While a number of groups have sought to tackle the ion yield issue in time‐of‐flight secondary ion mass spectrometry (TOF‐SIMS) by adding metals and other compounds that aid cationisation to the sample surfaces,^9^, ^10^, ^11^, ^12^ we have focussed on the possibility of enhancing proton positive ion yield using water cluster beams.^13^, ^14^ The idea was based on the observations of a number of groups that the presence of water, either adventitious or intentionally added, promoted the yield of protonated molecules and related secondary ions. It has been shown that there is a significant ion yield benefit to be obtained from the use of water clusters as primary ion beams in the analysis of bio‐organic molecules. This benefit is particularly significant for TOF‐SIMS if an instrument is used that can collect all the ions generated well beyond the static limit that previously constrained analysis using high energy small metal cluster primary ions.

Argon cluster beams can be used as very effective primary beams for the analysis of biological systems and it has been shown that they are optimally effective where the primary energy per argon atom, *E/n,* is below about 10 eV.^15^, ^16^, ^17^, ^18^, ^19^ Although molecular fragmentation falls below this energy, yield does too. Water cluster beams behave in a similar manner to argon at *E/n* ~ 10 eV; however, the yield of [M+H]^+^ ions rises significantly to a maximum at *E/n* ~ 3 eV, or a cluster size of about 7000 at 20 keV beam energy.^14^ The yield enhancement varies with the chemistry of the analyte. In the cases studied to date the increase is in the region of 10 to 100 times. There is also some evidence that the matrix effect is ameliorated, although this has still to be fully demonstrated.^13^ Studies with (D_2_O)_n_ cluster beams have shown that enhanced protonation in the low *E/n* regime does arise mainly from the water molecules in the cluster. The mechanism of water cluster ion yield enhancement is a matter of some speculation; however, it is possible to derive some insights by combining theory from both molecular dynamics (MD) and empirical considerations,^15^, ^20^, ^21^ with the observations from our experiments. On this basis it is suggested that in the impact site some type of concerted mechanism occurs between the energised water cluster and analyte molecules to enhance the protonation process, resulting in increased yields of [M+H]^+^ and related ions.

Tissue and cell imaging requires good ion yields to enable not only the majority species to be detected, but also the molecules that may be present in low concentration and yet may have important biological functions. The demands of spatial resolution exacerbate this requirement. Angerer *et al*. have recently shown that a 40 keV Ar_4000_ cluster beam (Note *E/n* = 10 eV) incorporating 8% CO_2_ enables the beam focus to be optimised and is optimum in delivering good ion yields of lipids and glycosides from tissue samples.^17^ Some earlier studies suggested that incorporating other molecules into argon clusters could also increase secondary ion yield. Winograd's group have shown that around 3% of methane in Ar_2000_ provides around 3 to 10 times increase in the ion yield from some molecules,^22^ while around 10% of CO_2_ increases the yields of some ions and also increases the stability and focus of the argon cluster beam. The present paper will demonstrate that doping argon cluster beams with water also enhances yield. Following up on this observation the study seeks to assess which of the beams that we have available is optimum for tissue imaging.

While metal cluster ion beams from liquid metal sources provide the most straightforward route to high spatial resolution (<300 nm being routinely attained), and although recent protocols have been advocated to extend their useful analytical range beyond the static limit, their use is still constrained by beam‐induced chemical damage.^23^ C_60_^+^ beams can be focused to sub‐1 µm, and for many situations can analyse well beyond the static limit; however, there are damage limitations for a number of systems and carbon deposition is observed at lower impact energy.^24^ Giant cluster beams offer the benefits of low damage and, in the case of water, higher yields; however, focusing the beam to sub‐micron resolution, although probably possible, is a challenge still to be overcome. This study will first compare the ion yields accessible from two model compounds using water‐doped argon clusters and argon and water clusters, and then compare 20 keV C_60_^+^, Ar_2000_^+^, water‐doped (H_2_O)Ar_2000_^+^ and (H_2_O)_6000_^+^ primary beams for mouse brain tissue analysis and imaging.

## Experimental

### Sample preparation

Model studies were carried out on three samples: trehalose, dipalmitoylphosphatidylcholine (DPPC) and mouse brain total lipid extract. Thin films of D‐(+)‐trehalose dihydrate (Sigma Aldrich, Gillingham, UK) were prepared by spin casting 30 μL of a 15 mM solution onto clean silicon at 6700 rpm. Thick films of 1,2‐dipalmitoyl‐*sn*‐glycero‐3‐phosphocholine (DPPC, Sigma Aldrich) were prepared from an 8 μM solution prepared with 0.003 g dissolved in 10:1 chloroform/methanol. Then 5 μL of this solution was dispensed onto clean silicon in dynamic mode at a rotation speed of 1700 rpm, accumulating a total of 100 μL to obtain a thick film for analysis. Brain total lipid extract was prepared from 50 mg of a snap‐frozen mouse brain stored at −80°C. The brain was homogenised with ice‐cold 1:1 MeOH/CHCl_3_ followed by sonication for 60 min. This produced a solution with clear separation of aqueous and organic phases and, since the lipids are contained in the aqueous phase, 10 μL of this solution was pipetted onto clean silicon and air‐dried prior to analysis with SIMS.

Serial sections from a wild‐type mouse brain were obtained using a cryo‐microtome (Wolfson Molecular Imaging Centre, The University of Manchester, Manchester, UK). The mouse brain was obtained following ethically approved procedures from the Faculty of Life Sciences, The University of Manchester. Each 8 µm thick section was thaw mounted onto clean silicon wafers with sagittal orientation. They were stored at −80°C and desiccated for 1 h at room temperature before analysis.

### TOF‐SIMS studies

TOF‐SIMS analysis was performed on a J105 *3D Chemical Imager* (Ionoptika Ltd, Chandler's Ford, UK) described in detail previously.^25^, ^26^ The J105 is equipped with a 40 keV C_60_^+^ primary ion beam (Ionoptika Ltd). A 20 keV gas cluster ion beam (GCIB) system (also supplied by Ionoptika Ltd) forms the basis of a second primary beam system that can provide either argon cluster or water cluster beams.^27^ As described in detail elsewhere, a temperature‐controlled water boiler source installed prior to the expansion chamber supplies water vapour to a heated (to prevent water condensation) Laval style nozzle with a 30 µm aperture for adiabatic expansion to form the neutral cluster beam in the expansion chamber, that is subsequently ionised by electron bombardment.^14^

Three cluster beams were used in these studies: dry argon, Ar_n_, clusters and pure water clusters, (H_2_O)_n_, and what is referred to as ‘wet’ argon or (H_2_O)Ar_n_ clusters. The latter beams are formed as follows. High‐pressure argon is passed over water in the boiler heated to a predetermined temperature to form an argon‐water mixture. This mixture then passes through the heated Laval nozzle into the expansion chamber to form a mixed water‐argon beam. Studies have shown that above a water partial pressure of about 1 bar, water cluster beams are formed in preference to argon cluster beams. This is probably partly because the nozzle temperature has to be close to 100°C to prevent condensation and consequently the argon clusters are destabilised, whereas because of stronger intermolecular forces the water clusters are stable. A boiler temperature of ~85°C and a nozzle temperature of 95°C result in a stable water‐doped argon cluster beam. Based on the expected water partial pressure of about 0.8 bar with an argon pressure of 14 to 16 bar, we estimate that the water composition is about 5%. To be certain of the composition a residual gas analyser in the beam line would be ideal; however, this was not available. (H_2_O)Ar_n_ cluster beams in the range *n* = 1000 to 6000 have been studied.

The first set of experiments aimed to assess the ion yield changes consequent on the application of the three cluster beams. The three model samples, the sugar trehalose, the lipid DPPC and a brain total lipid extract as described above, have been studied. Positive ion spectra after a primary ion dose of 5E11 ions cm^−2^ followed by a dose‐dependent study of spectral changes up to a dose of ~3E13 ions cm^−2^ were obtained for each sample using 20 keV water‐doped argon cluster beams (H_2_O)Ar_1000_ to (H_2_O)Ar_6000_ and compared with the data obtained previously using dry argon and water cluster beams in the same size range.^14^ As in this earlier study, in order to correct for any instrumental variations and to confidently compare ion yields between experiments using the different cluster beams, all ion signals were referenced to the total ion signal observed for each standard sample from a 20 keV C_60_^+^ analysis after a dose of 3E13 ions cm^−2^. The reference experiment was carried out before each series of argon or water cluster beam experiments.

### Tissue imaging

For the tissue imaging experiments a secondary electron microscope (SEM) image from a 300 copper mesh (Agar Scientific, Stansted, UK) was used to measure and optimise the lateral resolution of each primary ion beam. The lateral resolution was measured using a 100 µm aperture in the ion beam column, generating profiles from vertical and horizontal grid bars. The spatial resolution obtained for each beam was between 9 and 13 µm.

The whole cerebellum from different serial sections was analysed with the four primary ion beams for comparative studies. Each image contains 10 × 10 tiles. Further details are provided below. All the sections used for the analyses belong to the same stage of development of the brain to minimise chemical variation. The brain regions contain unique anatomical features that are easy to identify.

## Results and Discussion

### Model compounds

Our previous study demonstrated that (H_2_O)_n_^+^ beams give rise to a significant increase in the yield of secondary ions that rely on protonation for their formation. Because of the minimal damage caused by these beams, the yield increase at a dose of around 1E13 ions cm^−2^ *compared with static conditions* using C_60_^+^ or Ar_n_^+^ beams is dependent on analyte chemistry and ranged from around 100 to 1000 times.^14^ This enhancement occurred in the low energy per water molecule region, *E/n* of about 3 eV. In this energy range the yield from Ar_n_^+^ beams was very low. In the light of the work from the Winograd lab referred to above suggesting that doping argon clusters with other hydrogen‐containing molecules increased protonated analyte yields, a study of doping argon clusters with water has been carried out.^22^

**Figure 1:**
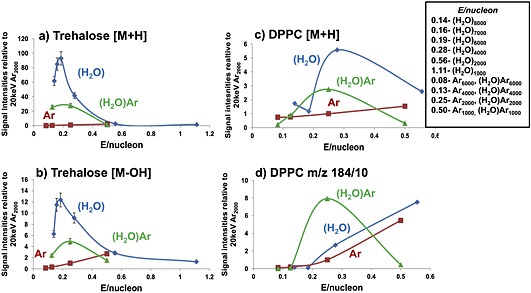
Comparison of the ion signals from Ar_2000_ using water (H_2_O) – blue; argon (Ar) – red; and wet argon (H_2_O) Ar – green; cluster beams as a function of *E/nucleon* at 20 keV for trehalose after an ion dose of 3E13 cm^−2^: (a) [M+H]^+^ *m/z* 343 and (b) [M+H–H_2_O]^+^ *m/z* 325; and DPPC after an ion dose of ~3E13 cm^−2^: (c) [M+H]^+^ *m/z* 734 and (d) the phosphocholine fragment ion *m/z* 184 (signal intensity/10).

As described in the Experimental section doping argon clusters with water has to be limited because of the tendency to preferentially form water clusters when the water partial pressure gets close to 1 bar. By keeping the water partial pressure at about 0.8 bar, doped argon cluster beams composed of between 1000 and 6000 atoms have been formed. Using these beams we have studied the ion yields from two of the model compounds, trehalose and DPPC, that featured in the previous comparison of pure water and argon cluster beams. In Fig. 1 the ion signals from the water‐doped argon cluster beams are compared with the previous data from pure argon and water beams after a dose of around 3E13 ions cm^−2^. The ion signals observed for two ions that rely on protonation from each compound are plotted as a function of the energy per nucleon, *E/nucleon,* in the cluster. It can be seen that (H_2_O)Ar_n_ increases the trehalose [M+H]^+^ ion signal compared with Ar_n_ by about 20× at *E/nucleon* 0.2 to 0.25 or (H_2_O)Ar_2000_. This enhancement is only a factor of 3 less than the maximum seen for the pure (H_2_O)_n_ beams at close to the same *E/nucleon*. The [M+H–H_2_O]^+^ fragment ion yield from trehalose also maximises at this point. The [M+H]^+^ signal from DPPC maximises close to *E/nucleon* ~ 0.25 for (H_2_O)Ar_2000_ at 3× that observed for pure argon, but 50% of the maximum seen from pure water beams. The phosphocholine *m/z* 184 fragment ion from DPPC falls continuously with water and argon cluster beams as *E/nucleon* decreases, whereas with (H_2_O)Ar_n_ the ion yield maximises at *E/nucleon* ~ 0.25. It is interesting and significant that the [M+H]^+^ maxima for water clusters and (H_2_O)Ar_n_ clusters align rather well at an energy per nucleon of about 0.2 eV. At this beam energy there is a very small yield of [M+H]^+^ ions from pure argon clusters, so the addition of this small amount of water has had a remarkable effect and seems to produce results similar to those from pure water clusters.

### Deuteration studies

**Figure 2:**
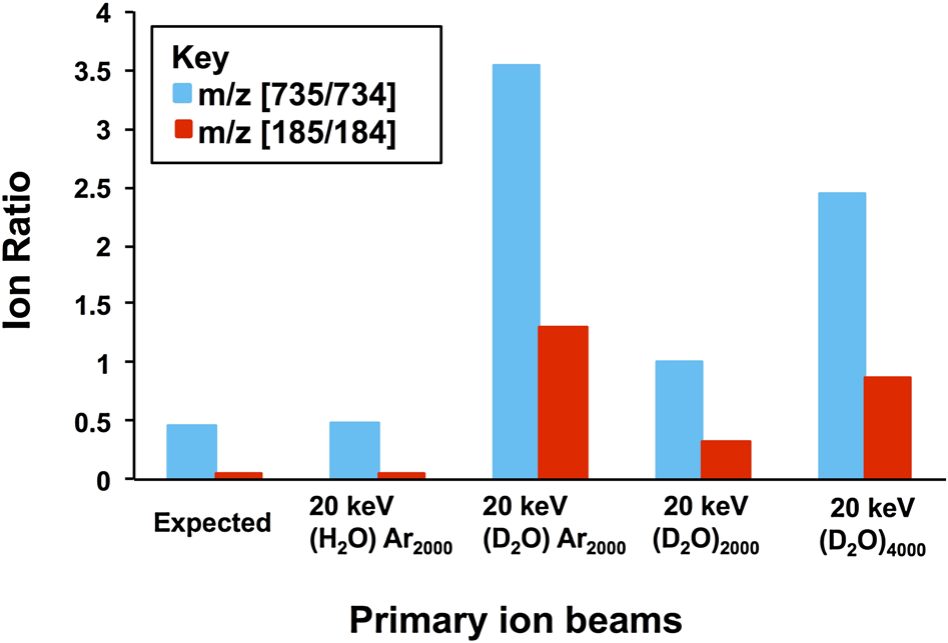
Ion signal ratios for *m/z* (735/734) and *m/z* (185/184) from DPPC obtained with 20 keV (H_2_O)Ar_2000_; (D_2_O) Ar_2000_; and 20 keV (D_2_O)_2000_ and (D_2_O)_4000_ cluster primary ion beams at a dose of 5E11 ions cm^−2^. The ‘Expected’ ratios are those that would arise from the ^13^C contribution.

To explore the water doped argon effect further, a study using D_2_O to dope the argon was carried out. Exactly the same procedure was used as to form the (H_2_O)Ar_n_^+^ beam. Only the Ar_2000_ cluster was studied and the results were compared with those observed using 20 keV (H_2_O)_2000_^+^_,_ (D_2_O)_2000_^+^ and (D_2_O)_4000_^+^ from our previous study.^14^ The ion signal ratios of *m/z* 185/184 and 735/734 observed from DPPC are presented in Fig. 2. After appropriate allowance is made for the ^13^C‐isotopic contribution to the *m/z* 735 and 185 ions, it can be concluded that there is significantly more deuteration with (D_2_O)Ar_2000_^+^ than with pure (D_2_O)_2000_^+^. The level of deuteration is in fact closer to that observed for (D_2_O)_4000_^+^. This conclusion is also confirmed by the trehalose deuteration experiments (data not shown). While at first sight it might be thought that there would be a different mechanism involved in protonation by (H_2_O)Ar_n_^+^ from that with pure water clusters, the fact that the yields maximise at the same *E/nucleon* value of about 0.2 and that the extent of deuteration from (D_2_O)Ar_2000_ is closer to that observed from (D_2_O)_4000_ where the *E/nucleon* is 0.25 suggest that a common mechanism is in operation.

In our previous study we concluded that when *E/n* (where *n* is the number of argon atoms or water molecules) was around 10 eV, or a cluster size of 2000 at a beam energy of 20 keV, water molecules behaved rather similarly to argon atoms. They sputtered off analyte molecules, but there was little enhancement of the ions from the protonated analytes. However, at low *E/n* it was suggested that a concerted mechanism came into play in which, because of their hydrogen‐bonded stability, large water clusters largely retained their cluster geometry and interacted with analyte molecules in the impact zone with sufficient activation to enhance protonation, but with minimal molecular fragmentation. In the case of water‐doped argon it appears that the mechanism may be very similar. When the data is viewed in terms of *E/nucleon*, both the ion yields and the extent of deuteration maximise at a beam energy in the region of 0.2 eV/nucleon. MD simulations have shown that it is highly likely that at these low energies the impacting cluster maintains a good deal of its structure.^20^, ^21^, ^28^ The sputter rate and fragmentation of analyte molecules are both very low as was shown in our previous paper.^14^ Delcorte *et al*. have explored in some detail the sputter yield and fragmentation of molecular species as a function of *E/nucleon* from large cluster impacts.^28^, ^29^ This work suggests a mechanistic change at an *E/nucleon* of about 1 eV. *Above* this energy the sputter yield varies linearly with beam energy. The sputtered mass depends only on the beam energy and not its nuclearity and much of the energy of the projectile is deposited in a 2–4 nm depth in the centre of the impact zone in a time <100 fs. A greater yield of molecular fragments is expected because of the high energy density. As a consequence for organic analytes higher yields of free hydrogen could also be expected that might contribute to protonation as the hydrogen flows out to the lower energy periphery of the emission zone. On the other hand where the *E/nucleon* is significantly *below* 1 eV, the rate at which energy is deposited in the substrate is significantly slower. The time for the projectile to move 2 nm increases from about 100 fs to more than 300 fs at an *E/nucleon* of 0.2 eV. Very much less energy is deposited in the central impact region so low levels of molecular fragmentation or generation of free hydrogen are predicted. The energy deposited in the outer, lower energy rim of the impact zone is less than the binding energy of the molecules, so the outer rim is where most of the intact molecular emission is expected. These conclusions are supported by recent MD studies of the impact of large argon clusters on octane and β‐carotene by Postawa *et al*.^30^

These ideas align very well with our observations for water and water‐doped argon beams when the *E/nucleon* ≥0.5 eV. Their behaviour is very similar to that of argon clusters, probably because the time scale of energy deposition is so fast there is no time for the hydrogen from the water molecules to react with the analyte molecules. However as the *E/nucleon* declines, enhanced protonation as a consequence of the presence of water increases. For the samples that we have studied proton enhancement seems to maximise close to *E/nucleon* ~0.2 eV. Delcorte's ideas suggest that impacting cluster particles are largely retained in the impact site over many 100s of fs and the molecules are moving more slowly, providing time for protonation to occur as the molecules are emitted from the surface. The fact that there is an ion yield maximum probably reflects that the sputter yield is becoming very small and although protonation is favoured by the low energy impacts, reaction rates will also fall as the impact activation falls.

It is intriguing that the (H_2_O)Ar_2000_ beam with only about 5% water content nevertheless provides between 30 and 50% of the yield enhancement of a pure (H_2_O)_6000_ beam. It may be speculated that interaction between the 10 eV argon atoms and water molecules in the impact site leads to more dissociation of the water molecule resulting in a higher density of free hydrogen leading to relatively higher protonation yields.

### Brain extract studies

**Figure 3:**
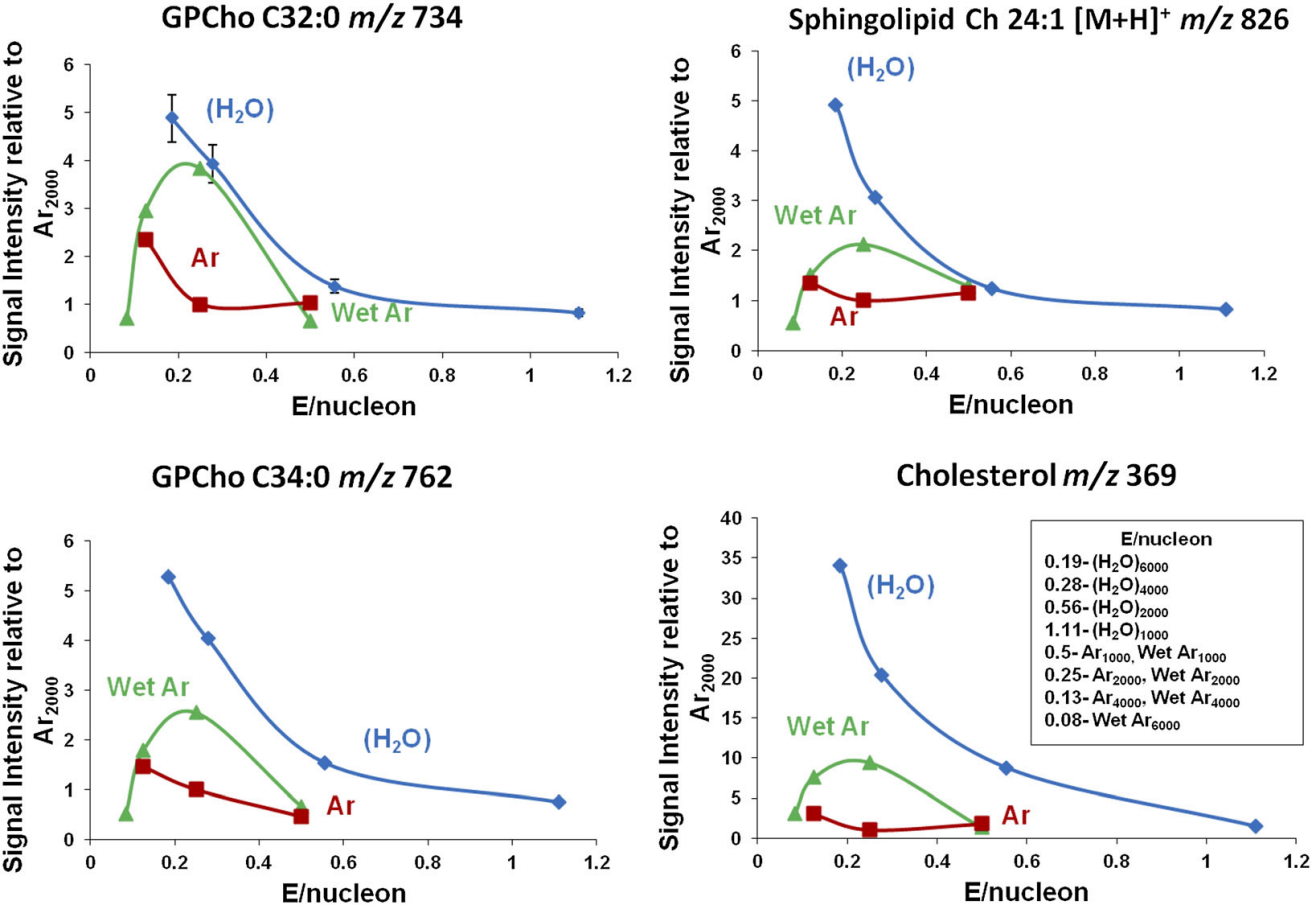
Comparison of the ion signals from mouse brain extract relative to those observed from Ar_2000_ using water (H_2_O)_n_ – blue; argon Ar_n_ – red; and water‐doped argon (H_2_O)Ar_n_ – green; cluster beams as a function of *E/nucleon* at 20 keV for the [M+H]^+^ ions from the phospholipids GPCho C32:0 at *m/z* 734; GPCho C34:0 at *m/z* 762; sphingolipid Ch 24:1 at *m/z* 826; and the cholesterol [M+H–H_2_O]^+^ ion at *m/z* 369.

Having confirmed the efficacy of (H_2_O)Ar_n_^+^ beams in increasing protonated secondary ion signals on model compounds, a study was carried out on homogenised mouse brain tissue to confirm whether the benefits of water and water‐doped argon beams were observed from this ‘real’ biological sample. The variation of secondary ion signals from the cholesterol [M+H–H_2_O]^+^, *m/z* 369 ion, that requires protonation of the parent molecule followed by loss of H_2_O, together with the [M+H]^+^ ions from three phospholipids observed from the brain extract have been monitored as a function of cluster beam size and hence *E/nucleon* for the three beams. The results are shown in Fig. 3. The ion signal behaviour follows very closely that observed for the model compounds. The lipid yields from Ar_n_^+^ beams are low, particularly for cholesterol. 20 keV C_60_^+^ was also tested (data not shown) and the observed yields were higher for cholesterol, but lower for the other lipids. The [M+H]^+^ signals maximise at *E/nucleon* ≈ 0.2 eV, using the pure water beams at a cluster size of about 6000, and with (H_2_O)Ar_n_^+^ at a cluster size of 2000. It seems particularly significant that the signal for cholesterol is very low under the pure argon cluster beam whereas it is 10× higher under (H_2_O)Ar_2000_^+^ and 35× greater under (H_2_O)_6000_^+^. The phospholipid enhancements are close to those observed for pure DPPC. Using the sputter yield plot (Fig. 1 from our previous paper^14^) it is possible to show that the number of molecules sputtered using 20 keV (H_2_O)_6000_^+^ is somewhat less than 50% of the number sputtered by 20 keV Ar_2000_^+^. Combining this fact with the observed ion signal enhancements suggests that the (H_2_O)_6000_^+^ beam results in an ion yield enhancement ranging from 10 to >100x, while enhancements due to (H_2_O)Ar_n_^+^ lie in the 4 to 10x range.

### Tissue imaging

On the basis of the brain extract studies the performance of the 20 keV C_60_^+^, Ar_2000_^+^, (H_2_O)Ar_2000_^+^ and (H_2_O)_6000_^+^ beams was investigated for tissue imaging. First the optimum beam focus and spot size were characterised for the argon and water beams using a metal grid structure. The beam focus obtained and used for 20 keV C_60_ was 3 µm; for Ar_2000_ was 13 µm; for (H_2_O)Ar_2000_, 11 µm; and for (H_2_O)_6000_, 9 µm. The presence of water in the cluster beams seems to enable a tighter focus to be obtained.

**Figure 4:**
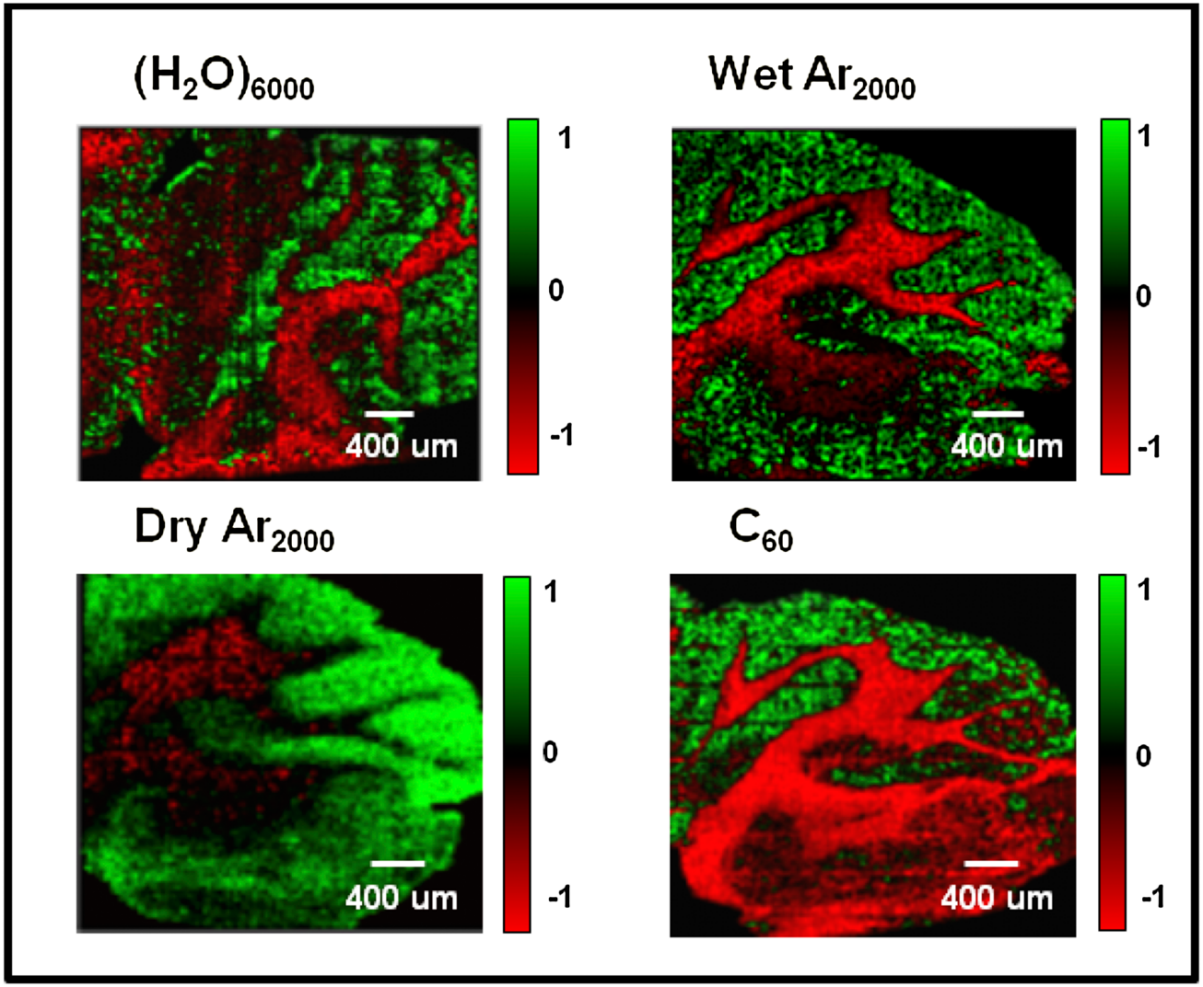
Imaging principal component analysis (PCA) images from the mouse brain cerebellum using four different cluster ion beams: 20 keV (H_2_O)_6000_^+^, 20 keV (H_2_O)Ar_2000_^+^, 20 keV Ar_2000_^+^ and 20 keV C_60_^+^. These images show the clear separation between the grey and white matter of the brain. The cerebellum is located above the medulla and consists of a cortex of grey matter (green) and a white matter (red) central core. These images show anatomical features such as the foliation pattern of the cerebellum lobules and the cerebellar nuclei above the medulla. The white matter fibre tracts are also easy to recognise due to their high content of cholesterol. The area covered by each image is 4 mm × 4 mm with a total ion dose of 1 × 10^12^ ions cm^−2^.

Secondary ion images of the cerebellum and medulla areas of the mouse brain were generated from 10×10 image tiles of the 400 µm field of view, each of these composed of 32×32 pixels. Each tile was exposed to 1E12 primary ions cm^−2^. Imaging SIMS generates multidimensional data that can be reduced to a small number of relevant dimensions with multivariate analysis techniques. Imaging principal component analysis (PCA) analysis was applied to the images obtained using the four beams. A region of interest (ROI) was drawn manually around the tissue area to avoid any interference of inorganic ions from the silicon substrate. PCA was carried out using MATLAB (The MathWorks Inc., Natick, MA, USA) calculating eight principal components (PCs) that represent most of the spectral variance within the dataset. The PCA results are displayed as image information by the scores from each PC. Each score is a density colour plot that contains the relevant information from each pixel (or spectrum). The resultant scores from the PCA analysis of the images are shown in Fig. 4. From the score images in this figure we can appreciate that pixels with similar chemistry are displayed in the same colour. Positive loadings from each PC are visualised as green pixels whereas the negative loadings are coloured in red.

The scores displayed in Fig. 4 were selected according to the clarity in which they exhibit the separation between the grey and white matter of the brain. Dependent on the ion beam used this separation was highlighted by different PCs. The score image from PC1 provided the clearest separation for C_60_^+^. The score images from PC2 and PC3 show the clearest separation for Ar_2000_^+^ and (H_2_O)_6000_^+^, respectively, whereas PC5 is clearest for (H_2_O)Ar_2000_^+^.

PC loadings plots corresponding to scores plots shown in Fig. 4 indicate as expected that one of the principal sources of variance between white and grey matter is the cholesterol [M+H–H_2_O]^+^ ion at *m/z* 369. This ion features very strongly in the white matter and is very weak in the grey matter under the Ar_2000_^+^ and C_60_^+^ beams. Other lipid peaks are evident in the loadings of white and grey matter although significantly stronger in the grey matter. The detection of cholesterol in brain tissue using TOF‐SIMS has been a matter of some discussion and provided differing results.^31^, ^32^, ^33^, ^34^ It has been shown that cholesterol can move to the surface and even be lost under vacuum conditions, although analysis at low temperatures inhibits this effect. Depth profile studies of brain tissue in this laboratory some years ago showed that the cholesterol level in white matter fell dramatically with depth when the analysis was carried out at room temperature, whereas when the tissue was held at low temperatures (~−100°C) while lower to start with, the cholesterol level did not change significantly with depth, although other lipid signals did fall and protein‐related amino acid peaks rose.^35^ A recent study by Angerer *et al*. in which brain tissue at room temperature was exposed to trifluoroacetic acid (TFA) prior to analysis has suggested that TFA may remove cholesterol from the surface of tissue allowing previously undetected molecules to be observed. TFA also has the benefit of increasing [M+H]^+^ ion yields quite significantly.^36^ The effects seem to be most noticeable in the white matter and granular regions. However, we know that the lipid content of the myelin‐rich white matter is between 55 and 70% of the dry weight and is composed of about 27% cholesterol and 45% phospholipid, whereas the lipid content of the more cellular grey matter, which is around 35% of the dry weight, is about 22% cholesterol and 60 to 70% phospholipid.^37^ Thus there is significant cholesterol content *throughout* the white and grey matter.

**Figure 5:**
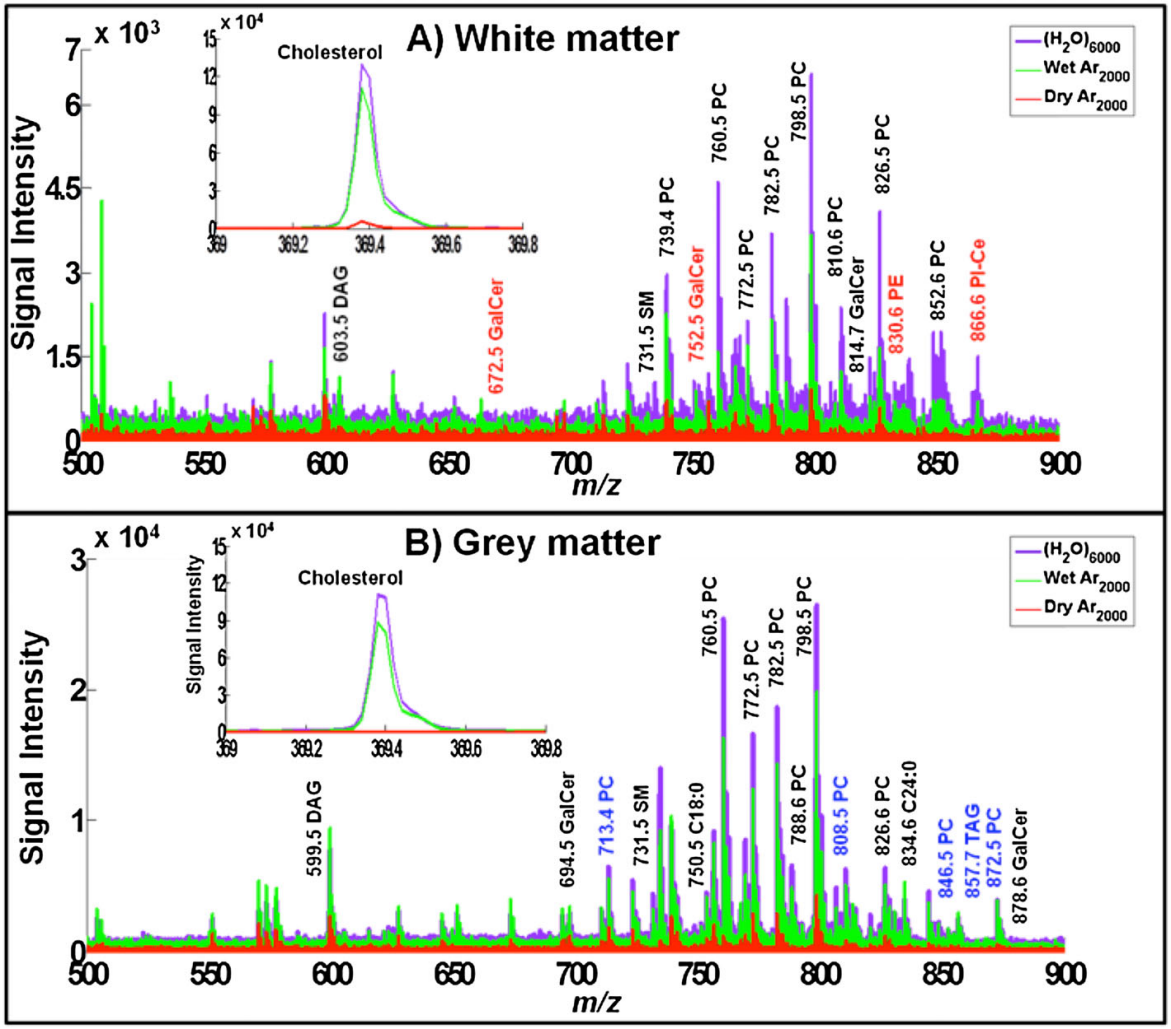
Spectral comparison from a 400 × 800 µm area between (A) white matter and (B) grey matter. The mass regions shown are *m/z* 500–900 and 369–370 where most lipids and cholesterol can be observed. Each set contains the overlay spectra of three beams: Ar_2000_^+^ in red, (H_2_O)Ar_2000_^+^ in green and (H_2_O)_6000_^+^ in purple. Some of the lipid peaks have been labelled for comparison purposes. Black labels are the peaks observed in both grey and white matter; red labels are observed only in white matter and those with blue labels are exclusively observed in grey matter.

In the present study we have compared the molecules detected from the white and grey matter regions using the three large cluster beams. To do this, using each cluster beam PC image we have selected regions that are, as far as possible, exclusively white or grey matter of the same geometric area and summed spectra have been generated from each area. The resulting spectra from these white and grey matter areas are overlaid in Fig. 5 and a selection of representative peaks is listed and assigned in Table 1 together with the signal enhancements observed in white and grey matter using the (H_2_O)Ar_2000_^+^ and (H_2_O)_6000_^+^ beams over the signals observed using Ar_2000_^+^ (columns 5 to 8). The main mass range displayed is the intact and large fragment lipid region, *m/z* 500 to 900. The inset shows the cholesterol *m/z* 369 region. Focusing on the latter region first, it can be seen that cholesterol is detected in the white matter using Ar_2000_^+^ but, as was observed for the brain extract, it is enhanced by around 20× using the water‐containing beams. In contrast, cholesterol is hardly detected in the grey matter using Ar_2000_^+^, as is also found under C_60_^+^ bombardment. In contrast, the cholesterol *m/z* 369 ion is clearly observed in the grey matter with the water‐containing beams approaching a similar intensity to that in the white matter, having been enhanced by 100 to 200×. Although in most SIMS images of brain tissue cholesterol normally only shows up in the white matter,^38^ this result does accord qualitatively with what is known of the grey matter composition.^37^

**Table 1:**
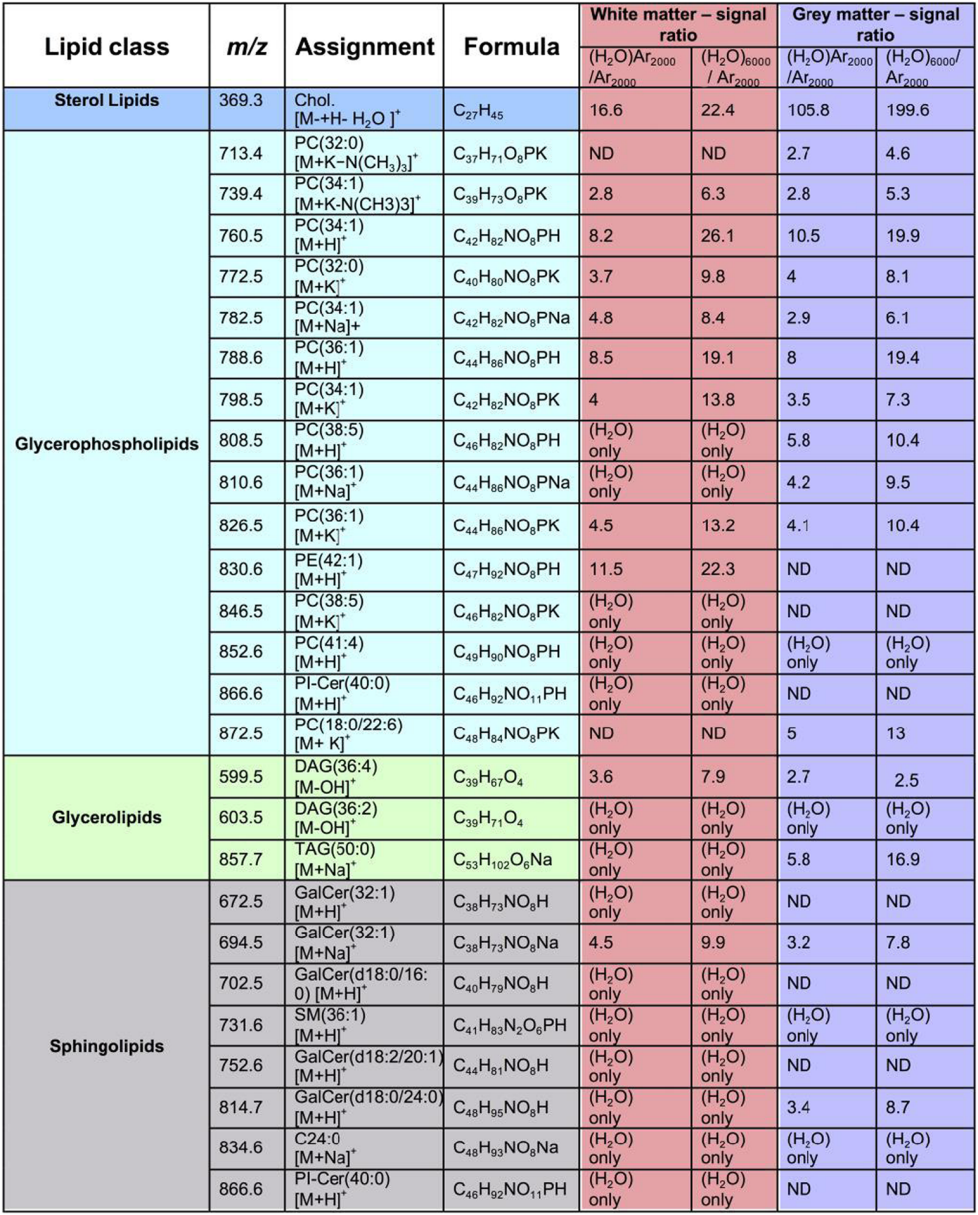
A selection of representative positive ions observed in white and grey matter in Fig. 5. Assignments based on mass measurement to 10 ppm using literature data.^36^, ^42^, ^43^ Ratios of ion yields observed in white and grey matter using the water‐containing cluster beams to those ions detected using Ar_2000_ are presented. Some ions are only detected using the water‐containing beams, labelled (H_2_O) only

**(H_2_O) only** – this peak was only observed with (H_2_O)Ar_2000_ and (H_2_O)_6000_; **ND** – Not detected. Phosphatidylethanolamine (PE), Phosphatidylcholine (PC), Phosphatidylinositol (PI), Galactosylceramide (GalCer), Sphingomyelin (SM), Diacylglyceride (DAG), Triacylglyceride (TAG), Ceramide (Cer), Cholesterol (Chol)

**Figure 6:**
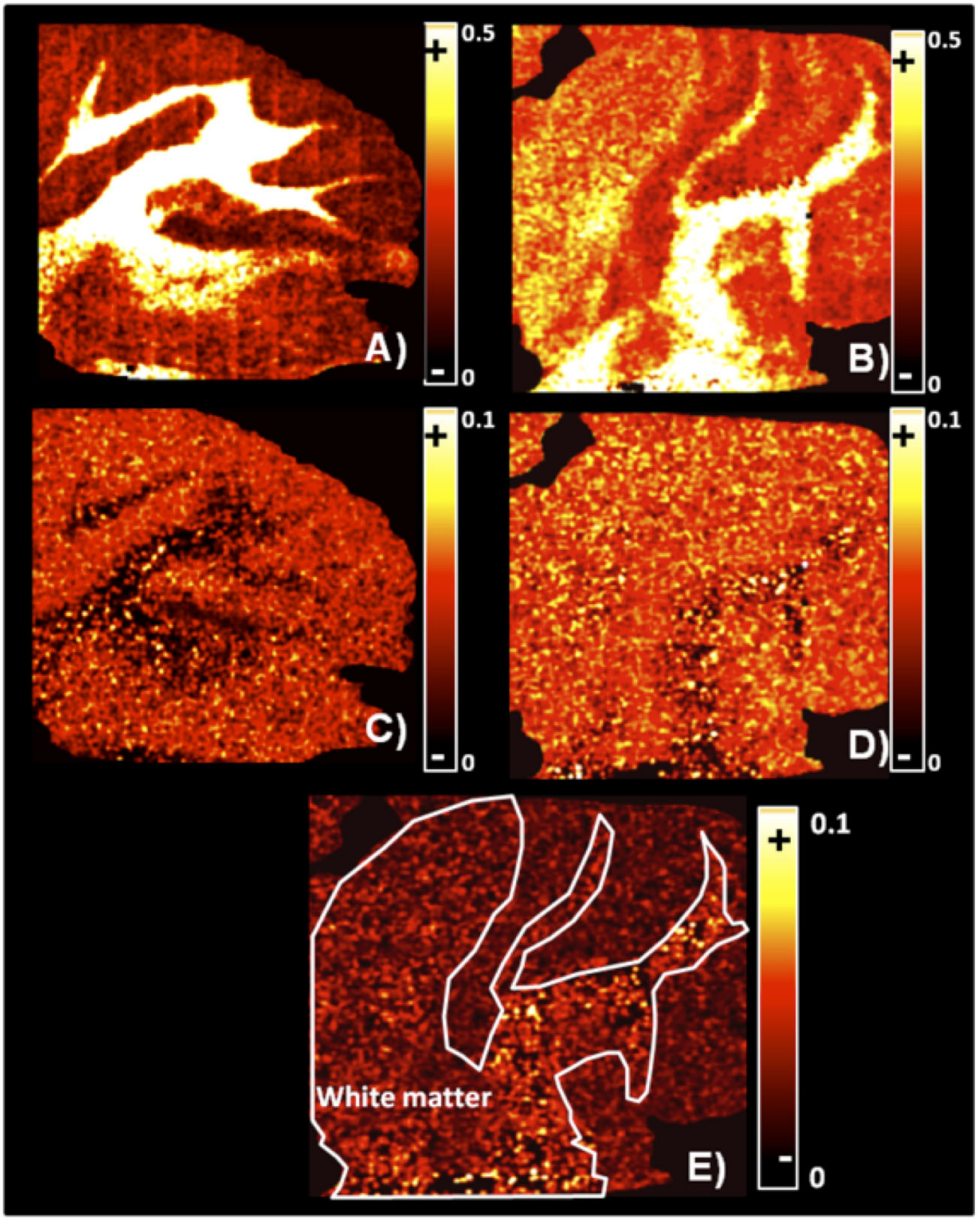
Sum‐normalised ion images from mouse brain generated by selecting a region of interest excluding the substrate (field of view = 4 × 4 mm): (A) (H_2_O)Ar_2000_ and (B) (H_2_O)_6000_ single ion images from cholesterol [M+H–H_2_O]^+^ at *m/z* 369.3 distributed across white and grey matter of the brain. (C) (H_2_O)Ar_2000_ and (D) (H_2_O)_6000_ PC36:1 [M+H]^+^ single ion images at *m/z* 788.6 co‐localised mainly in grey matter. (E) Addition of single ion images with (H_2_O)_6000_ from sphingolipids located in the white matter: GalCer(32:1) at *m/z* 672.5; GalCer(d18:0/16:0) at *m/z* 702.5 and GalCer(d18:2/20:1) at *m/z* 752.5. A white line was drawn around the white matter as a reference. (In each image the ion yield for the specified ion in each pixel was normalised to the total ion yield for that pixel. The images were then scaled to display better contrast and smoothed to highlight the specific anatomical features.)

In the presence of phospholipids the ionisation of many molecules is suppressed in SIMS and other mass spectrometries.^38^, ^39^ It is known that phospholipid [M+H]^+^ ion formation is enhanced by the presence of cholesterol.^40^ These effects may be due to the relative gas‐phase basicities of the compounds involved. The observation of clear phospholipid signals using Ar_2000_^+^, but the absence of cholesterol in cellular grey matter regions, may be a consequence of a matrix effect due to the greater proportion of phospholipids in this region. The fact that the water‐containing beams reveal the presence of cholesterol would suggest that there might be competition for protons between the cholesterol and the other lipids in grey matter. A model study in which a mixed film of cholesterol and DPPC was analysed does indeed show that the cholesterol [M+H–H_2_O]^+^ ion formation is quite dramatically suppressed (>10×) relative to the yield from the pure compound by the presence of the phospholipid using C_60_^+^ and dry Ar_2000_^+^ (data not shown). However, under (H_2_O)Ar_2000_^+^ and (H_2_O)_6000_^+^, the suppression is largely lifted as can also be seen in the *m/z* 369 ion images shown in Figs. 6(A) and 6(B). Although the cholesterol intensity is highest in the white matter, using the water‐containing beams there is significant intensity across the grey matter regions too. Thus, we can conclude that the absence of a cholesterol peak in grey matter using C_60_^+^ and Ar_2000_^+^ can be largely attributed to matrix ion suppression effects that are lifted in the presence of water in the cluster beam. It is, however, intriguing that although the overall relative cholesterol/other lipid composition of the two regions are not too dissimilar the matrix effect is seen strongly in the grey matter but not in the white. Earlier studies have shown that cholesterol can move at room temperature under the influence of the vacuum such that depth profiles do not reflect the true composition variation with depth.^38^ However, this does not seem relevant here. Jones *et al*. showed that the matrix effect suppression effects must be attributed to events at the surface or within the sputtering process.^41^ Thus, the physical structure of the two regions may also play a role in mediating the matrix effect. Grey matter is composed of numerous cellular structures so the phospholipids and cholesterol may be in close proximity, while white matter is composed of long‐range mylenated tracts or fibres where the physical proximity is rather different.

Turning to the other lipids detected in white and grey matter, many are very significantly enhanced under the water‐containing beams; however, it is also evident that there are many ions that are not visible under the pure Ar_2000_^+^ and C_60_^+^ beams that show significant intensity under pure water and water‐doped argon beams. This offers the prospect of detecting and imaging not just the compounds present at high concentration, but also the minor components that frequently have important biological function. Using our J105 instrument we can also increase the ion signal collected by increasing the ion dose and accumulating all the ions, although of course this extends the timescale of the experiment.

The spectra shown in Fig. 5 are complex and it is not the purpose of this paper to assign and discuss all the peaks. However, it should be noticed that the overall intensity of the peaks in the lipid region in the white matter is 2 to 4× less than in the grey matter, presumably reflecting the greater proportion of these lipids in grey matter. Focusing on the selection listed in Table 1 some other general observations can be made. The signal ratios in columns 5 to 8 of Table 1 provide a semi‐quantitative measure of the degree to which some lipid ions in the *m/z* range 500 to 900 are enhanced by the water‐containing beams in both white and grey matter. The [M+H]^+^ ions detected using Ar_2000_^+^ in both white and grey matter; for example, PC(34:1) at *m/z* 760.5 and PC(36:1) at *m/z* 788.6, are enhanced by 5 to 10× by (H_2_O)Ar_2000_^+^ and using the (H_2_O)_6000_^+^ beam by around 20×. These enhancements are in line with expectations arising from the model compound and brain extract studies reported above. Single ion images using the *m/z* 788.6 ion in Figs. 6(C) and 6(D) suggest that the detection of this ion is favoured from grey matter using the (H_2_O)Ar_2000_^+^ beam, whereas using the (H_2_O)_6000_^+^ beam it also shows up at a similar yield in the *white* matter. Perhaps a matrix effect is operating to inhibit PC protonation in white matter that is lifted by the water beam. As mentioned above the differing chemistry and physical state of the two regions may play a complex role in influencing ion formation.

There are quite a number of [M+H]^+^ ions that are detected using the water‐containing beams but not when using the pure Ar_2000_^+^ beam, e.g. PC(38:5) in white matter and PC(41:4) in white and grey matter. In the white matter we also see some very strong enhancements of between 40 and 140× for ceramide‐containing ions at *m/z* 752.5, 814.7 and 866.6. The signals from these three ions have been summed and an ion image using the water beam generated in Fig. 6(E). Galactoceramides are important components of myelin and are necessary for its function and stability.^44^ They also perform a function in signal transduction. It is significant that the water‐containing beams enable these molecules to be clearly seen and identified, because although they have been observed using MALDI, they have been detected less frequently with TOF‐SIMS.^45^ The study mentioned above using TFA exposure has suggested that the removal of cholesterol from the surface facilitates the detection of galactoceramides.^36^

The other general observation is that not only are [M+H]^+^ ion yields increased under the water‐containing beams, but the [M+Na]^+^ and [M+K]^+^ yields also seem to be increased significantly. Table 1 shows that enhancements of between 3 and ~10× are observed; e.g., the [M+Na]^+^ ion of PC(34:1) at *m/z* 782.5 and the [M+K]^+^ ion of PC(18.0:22.6) at *m/z* 872.6. At first sight this is puzzling. However, if the mechanism of ion formation is a concerted process involving the impacting water‐containing cluster in the emission zone, it is perfectly possible that the presence of water and protons could mediate the exchange and attachment of alkali cations to the departing molecules. Such processes are well known in the aqueous biological environment of lipids.^46^, ^47^ Thus for some molecules, water‐containing beams may be beneficial for more than just proton attachment. This an area that merits further study.

It is clear that water‐containing beams offer a real ion yield and matrix effect benefit over pure argon cluster beams and C_60_^+^. This would suggest that water‐containing beams should be used for imaging so that the yield per pixel is maximised and the matrix effect reduced. In most cases the pure water (H_2_O)_6000_^+^ beam delivers around 10× the ion yield from (H_2_O)Ar_2000_^+^ when allowance is made for the different sputter yields. Thus, pure water beams should be favoured.

In our previous report we highlighted the fact that higher energy beams would probably increase yield because of the possibility of generating larger clusters at the same *E/n or E/nucleon*. This was demonstrated in Supplementary Fig. S7 of our previous paper, where the yield at constant *E/n* was shown to increase with cluster size.^14^ One could envisage therefore that a 60 keV water cluster beam comprising 20000 water molecules could double the yield again. However, this effect does not operate with the water‐doped argon beams because the proportion of water in the argon cluster cannot rise above about 5% without the argon cluster breaking down. With a constant proportion of water in the beam we have shown that the yield from 20 keV (H_2_O)Ar_2000_^+^ is exactly the same as from 10 keV (H_2_O)Ar_1000_^+^ (data not presented); in other words, the yield from a water‐doped argon cluster is constant as a function of cluster size at the optimum *E/nucleon* of about 0.20 eV.

Overall therefore it would appear that the optimum beam for tissue imaging might be the pure water beam. In practical terms, however, with the prototype beam system used here there is a drawback in that using the cluster beams composed of 6000 molecules or more the water source lifetime is limited to less than 3 h. Large images can take longer to acquire than this. The water‐doped argon beam operates at much lower water temperature and the source lifetime is more than 8 h which makes large images a practical possibility, albeit with somewhat lower ion yields. It is expected that the lifetime issue from pure water beams will be addressed in future versions of the beam system. There is, however, a further difficulty with the cluster beams, namely the limitations in beam focus and hence spatial resolution. At 20 keV the limit is about 5 µm. Sub‐micron capability is frequently required for tissue and cell imaging. Higher energy beams offer the prospect of better beam‐focusing capability, perhaps into the sub‐micron regime. Together with the possibility of increased yield from pure water clusters this is obviously an instrumental development worth exploring.
